# Pancreatic Cancer: Nucleic Acid Drug Discovery and Targeted Therapy

**DOI:** 10.3389/fcell.2022.855474

**Published:** 2022-05-16

**Authors:** Hong Dai, Razack Abdullah, Xiaoqiu Wu, Fangfei Li, Yuan Ma, Aiping Lu, Ge Zhang

**Affiliations:** ^1^ Law Sau Fai Institute for Advancing Translational Medicine in Bone and Joint Diseases, School of Chinese Medicine, Hong Kong Baptist University, Kowloon, Hong Kong SAR, China; ^2^ Institute of Integrated Bioinfomedicine and Translational Science, School of Chinese Medicine, Hong Kong Baptist University, Kowloon, Hong Kong SAR, China; ^3^ Institute for the Advancement of Chinese medicine (IACM) .Ltd, Shatin, Hong Kong SAR, China; ^4^ Institute of Precision Medicine and Innovative Drug Discovery, HKBU Institute for Research and Continuing Education, Shenzhen, China

**Keywords:** pancreatic cancer, nucleic acid drugs, targeted therapy, aptamer-drug conjugates, antisense oligonucleotides, small interfering RNAs

## Abstract

Pancreatic cancer (PC) is one of the most lethal cancers with an almost 10% 5-year survival rate. Because PC is implicated in high heterogeneity, desmoplastic tumor-microenvironment, and inefficient drug-penetration, the chemotherapeutic strategy currently recommended for the treatment of PC has limited clinical benefit. Nucleic acid-based targeting therapies have become strong competitors in the realm of drug discovery and targeted therapy. A vast evidence has demonstrated that antibody-based or alternatively aptamer-based strategy largely contributed to the elevated drug accumulation in tumors with reduced systematic cytotoxicity. This review describes the advanced progress of antisense oligonucleotides (ASOs), small interfering RNAs (siRNAs), microRNAs (miRNAs), messenger RNA (mRNAs), and aptamer-drug conjugates (ApDCs) in the treatment of PC, revealing the bright application and development direction in PC therapy.

## 1 Introduction

Pancreatic cancer (PC) is one of the deadliest malignancies with poor outcomes, and over 90% of PC belongs to pancreatic ductal adenocarcinoma (PDAC) (Hidalgo, 2010). The overall 5-year survival rate for PC in 2020 is only 9%, which is predicted to cause the second mortality regarding cancer-related death in the upcoming decade (Siegel et al., 2020). It is known that early detection and treatment for patients largely contribute to the therapeutic effect on PC. Unfortunately, PDAC does not cause symptoms right away and it is hard to detect the disease early. Most of the patients are found to be in the advanced stages without efficient drug administration (Kleeff et al., 2016; Leroux and Konstantinidou, 2021). For now, surgical resection is still the main treatment method against resectable PDAC, and the chemotherapeutic strategy currently recommended for the treatment of PC has limited clinical benefit (Table 1) (Mcguigan et al., 2018). The updated reports have substantiated the important influence of intertumoral heterogeneity and tumor microenvironment on PDAC-based drug discovery (Sun et al., 2020). Thus, it is an urgent requirement to generate and develop more specific inhibitors by overcoming the aforementioned defects.

**TABLE 1:** Summary of advised treatment for patients with PDAC of different stages from RCTs.

	Tumor stage					
	Resectable	Borderline resectable and/or locally advanced unresectable	Metastatic			
Performance status	EOG 0/1 (2)	ECOG 0/1 (2)	ECOG 0/1	ECOG 0/1 (2)	ECOG 2	ECOG >2
1st line therapy	Surgery + gemcitabine and capecitabine	FOLFIRINOX or radiochemotherapy	FOLFIRINOX	Nab-paclitaxel + gemcitabine	Gemcitabine	Best care
2nd line therapy			Gemcitabine (+nab-paclitaxel)	Nal-irinotecan+ 5-FU (Or oxaliplatin + 5-FU)		
Median survival	26 months	∼12–18 months	∼11 months	∼9 months	∼6–8 months	<6 months

5-FU, 5-fluorouracil; FOLFIRINOX, folinic acid, fluorouracil, irinotecan, and oxaliplatin; nab, nanoparticle albumin-bound; nal, nanoliposomal.

The oncogene Kirsten Ras (*KRAS*) and tumor suppressor gene tumor protein 53 (TP53) could be detected through the genetic carcinogenesis of PDAC (Waters and Der, 2018). *KRAS* is the predominant isoform mutated in cancer and the isoform mutated exclusively in PDAC, driving the tumor initiation, progression, and metastasis. The increased desmoplasia, together with several cell types including cancer-associated fibroblasts (CAFs), pancreatic stellate cells (PSCs), muscle fibroblasts, and immune cells composes of the typical PDAC characteristics. Nucleic acid drugs consisting of ribonucleotide (RNA) or deoxyribonucleotide (DNA) have bright potential in gene inhibition, gene activation, immunity stimulation, and protein recognition. The nucleic acid drugs mainly include antisense oligonucleotides (ASOs) (Crooke et al., 2017), small interfering RNAs (siRNAs) (Castel and Martienssen, 2013), small activating RNAs (saRNAs), microRNAs (Hayes et al., 2014), mRNAs, ribozyme, aptamers (Kaur et al., 2018), and so on (Sullenger and Nair, 2016). From 1998 to 2020, ten oligonucleotides therapeutics have been approved by the FDA (Roberts et al., 2020). The abundant nucleic acid drugs provide diverse therapeutic strategies for overcoming the defects in intertumoral heterogeneity and tumor microenvironment. PC always suffers from multiple gene mutations, which are ideal targets for gene-silencing or alternative aptamer binding. Additionally, mRNA vaccines and related anticancer strategies have been paid more attention since the prevalence of COVID-19. The clinical trials data have demonstrated the promising prospects of mRNA vaccines against nonsmall cell lung cancer and advanced castration-resistant prostate cancer (Kubler et al., 2015; Huang et al., 2021). Notably, aptamers are single-stranded nucleic acids capable of binding to their targets through 3D complementarity, with high specificity and affinity as well as simple synthesizability and low immunogenicity. Importantly, much preclinical evidence has shown that aptamer-drug conjugates (ApDCs) are a promising therapy strategy against PC (Li F. F. et al., 2017). Theoretically, nucleic acid drugs based on tumor-specific genes and alternative tumor-specific antigens could be designed for any cancer. Thus, nucleic acid drugs might be a promising therapy strategy against PC.

In this review, we summarized the current progress of antisense oligonucleotides (ASOs), small interfering RNAs (siRNAs), microRNAs (miRNAs), messenger RNA (mRNAs), and aptamer-drug conjugates (ApDCs) in the treatment of PC and were detailedly explored to present a discussion and perspective of these therapies against PC.

## 2 Developing Nucleic Acid-Based Therapy of Pancreatic Cancer

Nucleic acid-based drugs can be classified into multiple types in terms of differences in chemical structure and functional mechanism, which include antisense oligonucleotides (ASOs) (Crooke et al., 2017), small interfering RNAs (siRNAs) (Castel and Martienssen, 2013), microRNAs (Hayes et al., 2014), mRNA, and aptamers (Kaur et al., 2018). From the first FDA-approved ASOs fomivirsen for clinical use in 1998 (Lundin et al., 2015), ten oligonucleotides therapeutics have been approved by the FDA as of January 2020, including six ASOs, two siRNAs, one RNA-based aptamer, and one mixture of ssDNA and dsDNA (Roberts et al., 2020). The dominating advantage of nucleic-acid based drugs is that their pharmacokinetics are based on the chemical entity, whereas their base sequence determines their target, allowing them to exhibit safety characteristic and potent pharmacodynamics.

### 2.1 Antisense Oligonucleotides

**FIGURE 1:**
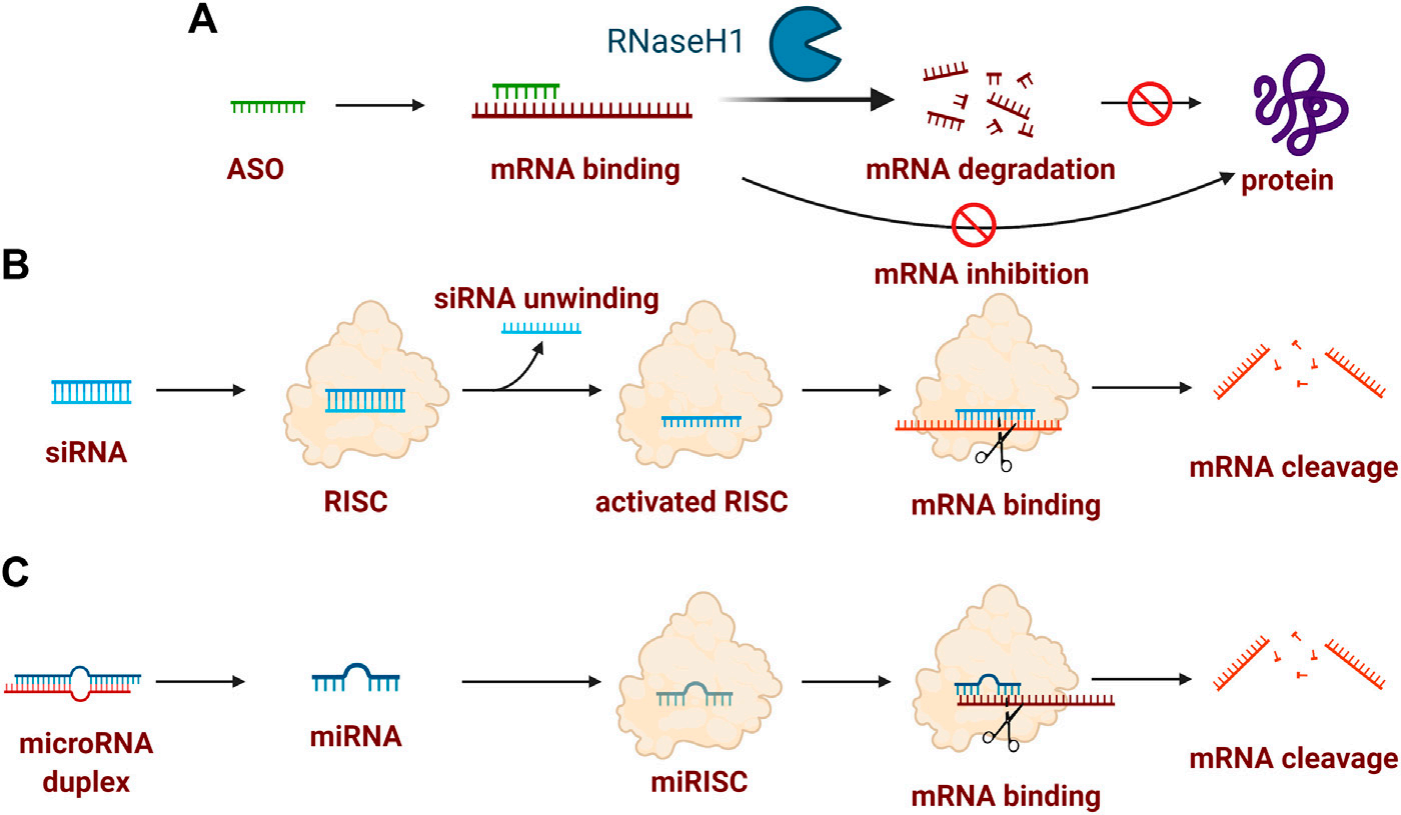
Typical mechanism of oligonucleotide drugs. **(A)** ASO-induced inactivation of mRNA could attribute to RNase H-dependent mRNA degradation or mRNA inhibition (also termed steric-blocker); **(B)** siRNA-indued mRNA cleavage contains the formation of siRNA-RISC complex, activated AGO2-RISC complex, followed by mRNA binding; **(C)** miRNA-induced mRNA cleavage contains the key formation of the miRISC complex for mRNA binding. Created with BioRender (License No: VV23OTLLHP).

ASOs are single-stranded RNA/DNA sequences containing usually 16–21 nucleotides in length and could specifically bind to RNA encoding the gene of interest *via* Watson–Crick base pair interactions in a sequence-specific manner. The inactivation of RNA induced by ASOs can be categorized broadly into two predominant termination mechanisms (Figure 1A): 1) RNase H-dependent: the destabilization by the formation of RNA/DNA duplex after recognition and binding to mature mRNA, followed by degradation by ribonuclease H1 (RNase H1), a ubiquitously expressed cellular endonuclease which specifically degrades DNA-RNA hybrids; 2) Steric-blocker: the internalization of ASOs lead to the binding to pre-mRNA, modulating translational arrest, alternative splicing, or disrupting recognition by splicing factors Bennett et al. (2017); Crooke et al. (2017); Shen and Corey (2018). RNase H-dependent manner is the dominant mechanism for ASOs and the following examples targeting PDAC are all based on this mechanism.

#### 2.1.1 Targeting *KARS* Point Mutations

The point mutations of the *KRAS* gene could be detected in 90% of human PCs, which indicates its important role in tumorigenesis. Therefore, targeting *KRAS* point mutations represents one potential way to treat pancreatic cancer. In 2001, a group investigated the invasive activity by one 17-mer phosphorothioate ASOs targeting *KRAS* point mutations in MIA PaCa-2, PANC-1, and BxPC-3 pancreatic cancer cells, and the results indicated that 17-mer ASOs could strongly inhibit the invasive activity of MIA PaCa-2, PANC-1 with aggressively mutated *KRAS* but not in BxPC-3 with a wild-type *KRAS* (Nakada et al., 2001). Additionally, ASOs targeting *KRAS* also inhibited the tumor growth and invasiveness of HaP-T1 (from the BHP-induced hamster PC model) in a dose-dependent manner, whereas mismatched ASOs were not effective in inhibiting invasion (Morioka et al., 2005). In 2013, the transfection of ASOs targeting *KRAS* point mutation by lipofectamine was investigated in human pancreatic carcinoma PC-2 cells. Further cell cycle assay showed majority of the cells were in the G1 phase and few were in the S phase, indicating the promotion of apoptosis of PC-2 cells induced by ASOs (Wang Y. X. et al., 2014). Another synthetic oligonucleotide with specific binding to *KRAS via* an ASOs-like mechanism, U1 Adaptors, also showed reduced *KRAS* mRNA expression up to 76% in MIA PaCa-2 cells. For the *in vivo* experiments, U1 adaptors were conjugated to tumor-targeting peptides such as cyclic arginylglycylaspartic acid (cRGD) resulting in the inhibition of tumor growth by averages of 68–93% (Carpizo et al., 2016).

#### 2.1.2 Targeting Heat Shock Protein

Heat shock protein 27 (Hsp27) is correlated to several pathological processes such as cancer and its high expression could be connected to chemotherapy-exposed PC. The modified ASO (termed as OGX-427) complementary to the Hsp27 gene was subtly designed and showed great inhibition of proliferation, induction of apoptosis, and improvement of gemcitabine chemosensitivity in MIA PaCa-2 cells both *in vitro* and xenografted in mice (Baylot et al., 2011). Despite gene suppression related to PC, it is necessary to consider the specific tumor microenvironment of PC especially PSCs. By construction of a tumor microenvironment-responsive nanosystem with PEGylated polyethylenimine-coated gold nanoparticles, all-*trans* retinoic acid and antiHSP47 ASO were codelivered to reprogram PSCs. Their results indicated that the nanosystem could induce PSC quiescence with a 79% downregulation of HSP47 protein and inhibit extracellular matrix (ECM) hyperplasia, therefore promoting drug penetration to pancreatic tumors and greatly improving the antitumor efficacy (Han et al., 2018).

#### 2.1.3 Targeting Vascular Endothelial Growth Factor

The vascular endothelial growth factor (VEGF) family of growth factors regulates angiogenesis and overexpression of VEGF in PDAC associated with a high angiogenesis density, improved metastasis, disease progression, and a reduced prognosis (Longo et al., 2016). One antiVEGF ASOs (AS-3) was designed and synthesized, which could normalize plasma VEGF and decrease neoangiogenesis, therefore indicating improved inhibition of tumor growth and metastasis as well as improved survival the in AsPC-1 xenograft nude mouse model. Notably, no ascites could be detected in animals treated with AS-3, demonstrating vascular permeability could be decreased by reducing VEGF expression in PC cells (Hotz et al., 2005).

#### 2.1.4 Targeting Bcl-2 Family and Transforming Growth Factor

Bcl-xL is an antiapoptotic factor of the B-cell lymphoma 2 (Bcl-2) family and its overexpression presence correlates with shorter patient survival. One anti-Bcl-xL ASO was shown to greatly inhibit PC cell growth and cause apoptosis with reduced Bcl-x_L_ protein levels. This Bcl-xL ASO also enhanced the cytotoxicity of gemcitabine in five PC cells (PANC-1, MIA PaCa-2, Capan-1, AsPC-1, and T3M4) (Xu et al., 2001). To improve the internalization into cells, anti-Bcl-xL ASO was further conjugated with antennapedia and this conjugate, together with irradiation as a supplement, significantly inhibited cell growth compared with ASOs alone and achieved meaningful results *in vivo* for radiosensitization of PC (Masui et al., 2006). Transforming growth factor-beta 2 (TGF-β2) regulates pivotal mechanisms including carcinogenesis, especially immunosuppression and metastasis within PC. Trabedersen (AP 12009) was one kind of ASO modified by phosphorothioate linkages, which targeted human TGF-β2 mRNA and was capable of reducing TGF-β2 secretion in Hup-T3 and PA-TU-8902 pancreatic cancer cells. This anti-TGF-β2 inhibitor ASO could greatly inhibit cell growth and absolutely block cell migration. Furthermore, this ASO was evaluated in the orthotopic mouse models experiments transplanted with metastatic PC, followed by the intraperitoneal (i.p.) treatment. This anti-TGF-β2 ASO could dramatically inhibit tumor proliferation as well as metastasis and angiogenesis (Stauder et al., 2004; Schlingensiepen et al., 2011). In a further phase II clinical study, a cohort of nine patients who were at the advanced PDAC stage and received intravenous trabedersen exhibited excellent safety as well as a promising overall median survival of 13.4 months (Helmut et al., 2012). However, no more clinical data are available to date.

#### 2.1.5 Targeting X-Linked Inhibitor of Apoptosis Protein and MicroRNA-21

AEG-35156 is a 19-mer phosphorothioate ASO targeting the caspase inhibitor X-linked inhibitor of apoptosis protein (XIAP) messenger RNA. XIAP is a representative sign to evade apoptotic cue-induced by chemotherapy in cancers including PC. Although the combination strategy of AEG35156 and gemcitabine showed promising results in preclinical models against PC, the combined therapy failed to show significant clinical activity in the phase I study in metastatic PC (Mahadevan et al., 2013). MicroRNA-21 is quite a popular cancer target, which has been demonstrated to correlate significantly with oncogenic initiation and progression. One study found that the combination of one antimicroRNA-21 ASOs (ASO-miR-21) and gemcitabine encapsulated by polyethylene glycol-polyethylenimine-magnetic iron oxide nanoparticles precoated with one target bullet fragment (scFv_CD44v6_) could downregulate oncogenic miR-21, thus leading to upregulation of tumor-suppressor genes programmed cell death protein 4 (PDCD4) and phosphatase and tensin homolog (PTEN) and the inhibition of aggressive invasion of PC cells *in vitro*. The combination strategy could induce much higher cell apoptosis when compared with a single dose of ASO or gemcitabine *in vitro*. Following *in vivo* data also demonstrated its good targeting ability as well as better efficacy in terms of tumor inhibition and liver metastasis (Li Y. Q. et al., 2017).

### 2.2 Small Interfering RNAs

Among the RNA interference mechanism existing in virtually all the eukaryotic cells, short double-stranded RNAs, i.e., small interfering RNAs (siRNAs), also adopt this ubiquitous pathway to degrade target gene mRNA and suppress its expression with high specificity. siRNAs usually consist of 21–23 nucleotides as a total length with two strands, one strand is the mRNA sequence called the sense strand and the other strand is called the antisense strand (Wittrup and Lieberman, 2015). Once inside the cells, siRNA could be recognized by the RNA-induced silencing complex (RISC) to formulate the siRNA-RISC complex. Then the sense strand could be cleaved by the cleavage enzyme argonaute-2 (AGO2) while the antisense strand and AGO2-RISC could formulate the activated AGO2-RISC complex. This complex is capable of searching and binding to an mRNA that contains a complementary sequence to antisense active strand, followed by the mRNA cleavage and inactivation of mRNA (Figure 1B) (De Fougerolles et al., 2007; Tatiparti et al., 2017).

Compared with ASO drugs, the transition from siRNA to drugs could go through similar problems but not limited to the degradation by nucleases, innate immune response, and most importantly inefficient transmembrane ability (Wittrup and Lieberman, 2015). Chemical modifications and the construction of a delivery system are usually adopted to overcome the above obstacles (Kanasty et al., 2013; Yin et al., 2014; Kim et al., 2016). For the two FDA-approved siRNA drugs Patisiran and Givosiran, 2′-O-methyl and 2′-fluoro substitution of 2′-OH ribose and part of thiophosphate linkages were taken (Adams et al., 2018; Roberts et al., 2020; Scott, 2020). Unlike highly-modified ASO, siRNA, in many cases, requires an appropriate delivery system given the larger size and the negative charges (Yin et al., 2014).

#### 2.2.1 Targeting *KARS* Point Mutations

Similar to ASO targeting *KRAS*, siRNAs also exhibited various effects in *KRAS* mutated PC. For example, K1/siRNA and K2/siRNA located at sites 194 and 491, respectively, were designed and transfected into MIA PaCa-2 cells, leading to increased apoptosis by inhibition of activated *KRAS* gene (Wang et al., 2005). But this siRNA expression cassette failed *in vivo* because of the short half-life and difficult transfection into cells. In 2007, another group designed and investigated three kinds of *KRAS* siRNA in the BxPC-3 (wild type-*KRAS* ^GGT^), Capan1 (*KRAS* ^GTT^), and PANC-1 (*KRAS* ^GAT^) cell lines. Their results indicated the corresponding *KRAS* siRNA induced a significant 80% inhibition of *KRAS* endogenous expression and cell proliferation at 100 nM drug concentration. *In vivo*, *KRAS* siRNA^GAT^ containing suppression of endogenous RNA (pSUPER) vector plus gemcitabine resulted in a prolonged survival rate of 64 days and dramatically reduced the mean tumor volume compared with single agents (52 days for gemcitabine, 45 days for *KRAS* siRNA^GAT^ containing pSUPER vector) (Rejiba et al., 2007). Another group investigated the combination effects of *KRAS* siRNA gene silencing activity and arsenic-mediated apoptosis with the aid of nanoparticles, a downregulation of *KRAS* mRNA and protein in PANC-1 cells (*KRAS* ^GAT^) treated with the combination therapy of up to 38.64 and 43.49% was achieved, respectively, resulting in enhanced inhibition of PC cell growth, migration, and invasion supplemented with the arsenic-mediated apoptosis. This combination therapy showed an improved efficiency of about 2-fold increase in the inhibition of tumor growth compared with the PBS group in the mouse models (Zeng et al., 2014). Ken-Tye Yang et al. (2015) developed one biodegradable charged polyester-based vector (BCPV) delivery system for *KRAS* siRNA within MIA PaCa-2 cells and found the growth, migration and invasion of the MIA PaCa-2 cells could be significantly inhibited. In mouse models, *KRAS* siRNA showed an improved accumulation for over 3 days and enhanced inhibition of tumor growth with the aid of BCPVs (Lin et al., 2017). Similarly, they also studied and demonstrated improved chemosensitivity of gemcitabine by BCPV-aided delivery of *KRAS* siRNA plus Notch1 siRNA in MIA PaCa-2 cells (Yang et al., 2017).

#### 2.2.2 Targeting Survivin and Nestin

Survivin is also one of the cancer-related targets with high expression including PC and evidence has shown radiation would lead to increased survivin mRNA expression in all cell lines. Therefore, antisurvivin siRNA was capable of preventing the radioresistance of PC cells including AsPC-1, SUIT-2, and PANC-1 (Kami et al., 2005). This reduction of survivin mRNA expression up to 90% through survivin-specific siRNA was also observed as well as pronounced morphological changes including enlargement of cells and multinucleation (Tsuji et al., 2005). The overexpression of nestin could also be observed in many cancers including PC, which is thought to be involved in cancer invasion and metastasis. Specifically, compared with monotherapy inhibition by gemcitabine, the combination of antinestin siRNA and gemcitabine exhibited enhanced cytotoxicity up to 80% of PANC-1 cells and 85% in PK-45H cells. Administration of antinestin siRNA significantly inhibited the tumor growth and metastasis compared to the negative control siRNA group in an orthotopic implantation model (Matsuda et al., 2016).

#### 2.2.3 Targeting Proto-Oncogene Tropomyosin Receptor Kinase A

The abnormal expression of nerve growth factor (NGF), together with its receptor proto-oncogene tropomyosin receptor kinase A (TrkA), is also connected with the aggressive and highly metastatic behavior of PC. Thus, TrkA-specific siRNA was designed and showed suppression of up to 83% of TrkA expression and kinase activity, and a much more increase of up to 5-fold of gemcitabine-induced, caspase-mediated apoptosis in PANC-1, MIA PaCa-2 and AsPC-1 (Liu et al., 2007). These results suggested TrkA might be a determinant of pancreatic adenocarcinoma chemoresistance. Xingyu Jiang et al. (Lei et al., 2017) developed a gold nanocluster-aided delivery system of NGF siRNA (GNC-siRNA) with a hydrodynamic diameter of about 70.2 nm. GNC-siRNA complex at 100 nM concentration showed 75% downregulation of NGF mRNA expression compared to 17% reduction of NGF mRNA expression by free siRNA, resulting in cancer growth and migration of PANC-1 cells. Most importantly, GNC-siRNA retarded siRNA degradation in serum conditions within 6 h resulted in a prolonged circulation lifetime of siRNA in blood and facilitated the siRNA internalization as well as the lysosomal escape. This GNC-siRNA also exhibited effective tumor regression in three pancreatic tumor animal models. Besides, the siRNA could be encapsulated by GNCs with the highest loading rate compared with other published data (226 mmol siRNA per g GNCs), which might serve as one promising delivery approach for PC treatment.

#### 2.2.4 Targeting Transforming Growth Factor and Bcl-2 Family

In terms of TGF-β signaling, its deregulation is usually related to tumor growth, invasion, and metastasis. Ski, as a TGF-β repressor, is an oncogenic protein and prevents related gene transcription. Anti-Ski siRNA-treated cells could restore TGF-β transcriptional activity and lead to a 5-fold decrease in tumor growth in nude mice (Heider et al., 2007). The combination of gene silencing of TGF-β1 (ppp-TGF-β) and immune activation *via* the cytosolic helicase retinoic acidinducible gene I (RIG-I) could remodel the immunosuppressive environment by recruiting CD8^+^ T cells and exert promising antineoplastic effects in PANC-1, MIA PaCa-2, and BxPC-3 cell lines as well as an orthotopic mouse model of PC. In addition, ppp-TGF-β with RIG-I activation also induced much more release of type I interferon and C-X-C motif chemokine ligand 10 (CXCL10), thus resulting in activation of CD8^+^ T cells and potent tumor cell apoptosis in orthotopic mouse model (Ellermeier et al., 2013). Other siRNAs targeting Bcl-2 mRNA (anti-Bcl-2 siRNA) were also proven to inhibit expression of the target gene *in vitro*, reaching maximum apoptosis of about 37.1% after 120 h at 10 nM concentration in YAP C PC cells. Within PC xenografts in male nude mice treated intraperitoneally, the tumor growth could significantly be inhibited but no more cytotoxicity could be observed on fibroblasts or normal tissues. However, the siRNA was rapidly metabolized and eliminated by the liver and excreted *via* the kidneys, thus explaining only a delayed growth of PC xenografts *in vivo* instead of strong suppression (Ocker et al., 2005).

#### 2.2.5 Other Targeting Therapy

Cyclooxygenase-2 (COX-2) correlates with the tumorigenesis of epithelial cancers including PC. The transfection of COX-2 siRNA (50 nM siRNA with 5 µl/2 ml Lipo) could downregulate about 70% of the expression of downstream COX-2 protein, further suppressing the vitality of Capan-2 cells but without causing significant cell apoptosis. However, anti-COX-2 siRNA could significantly inhibit tumor growth in subcutaneous xenografts nude mice (50 mm^3^/54.7 mg compared with 119.3 mm^3^/175.53 mg as the negative control) (Zhong et al., 2012). As PANC-1 cancer cells have higher nuclear factor-κB (NF-κB) and XIAP levels, antiNF-κB p65 siRNA could downregulate p65 protein and XIAP protein but not to a satisfying level. Further introduction of antiXIAP siRNA in combination with antiNF-κB p65 siRNA could effectively downregulate the expression of XIAP and p65 proteins and enhance the chemosensitivity of PC cells to gemcitabine (Cao et al., 2013).

Reprogramming tumor immune microenvironment (TIME) in PC plays a vital role because immune evasion of PC can include but is not limited to autophagy (Yamamoto et al., 2020). For instance, phosphatidylinositide 3-kinase γ (PI3K-γ) and colony-stimulating factor 1 (CSF-1)/colony stimulating factor 1 receptor (CSF-1R) pathways were thought to be involved in the infiltration and polarization of immunosuppressive cells. Therefore, dual blockade of PI3K-γ inhibitor NVP-BEZ 235 and antiCSF-1R-siRNA specifically targeting specific deactivated immune cells were developed. Therefore, the TIME could be reversed from inflammatory deactivation to inflammatory activation, further promoting a strong immune response and tumor growth *in vivo* (Li et al., 2020).

To facilitate the efficient delivery, one siRNA targeting polo-like kinase 1 (PLK1) was encapsulated by a novel nanosystem, which consists of cRGD modification on the surface of well-prepared ionizable lipid nanoparticles (GARP). This GARP/anti-PLK1 siRNA combination showed enhanced efficacy both *in vitro* and *in vivo* (Guo et al., 2021), which might give guidance on the implication against PDAC.

### 2.3 MicroRNAs

MicoRNAs, abbreviated as miRNAs, are single-stranded, short noncoding RNAs containing 20–24 nucleotides. MicroRNAs are indeed involved in all physiological signaling pathways, especially in mammals (Jansson and Lund, 2012; Dong et al., 2013). The mechanism of miRNAs-mediated gene silencing is achieved by directing Argonaute (AGO) proteins to related mRNA binding sites through a base-pairing mechanism, followed by the formation of the miRNA-induced silencing complexes (miRISCs). Therefore, the downstream deadenylation of the mRNA target could be catalyzed by the further formation of poly(A)-nuclease 2-poly(A)-nuclease 3 (PAN2-PAN3) and carbon catabolite repression 4–negative on TATA-less (CCR4-NOT) complexes, thus resulting in mRNA decapping and degradation (Jonas and Izaurralde, 2015; Gebert and Macrae, 2019). Notably, mRNA is usually cleaved by siRNAs and the translation is usually repressed by miRNAs depending on the degree of related complementarity (Figure 1C) (Siomi and Siomi, 2009).

#### 2.3.1 Targeting Upregulated MicroRNAs

A growing number of direct and indirect evidence has suggested a strong relationship between the deregulation of miRNA and PC. For example, miR-221/222, miR-376a, and miR-301 are overexpressed in pancreatic tumors (Lee et al., 2007; Xu et al., 2015). The overexpression of miR-21 can also be detected in PC cells and miR-21 contributes to cell proliferation, invasion, and chemoresistance (Moriyama et al., 2009). In addition, elevated levels of miR-200a and miR-200b (Li et al., 2010), miR-27a (Ma et al., 2010), miR-17-5p (Yu J. et al., 2010), miR-10b (Nakata et al., 2011), miR-155 (Mikamori et al., 2017), miR-196a and miR-1246 (Xu et al., 2017), miR-501-3p (Yin et al., 2019) can be detected in tumor cells, plasma, exosomes related to PC. However, in some cases, the downregulation of miRNA could also be observed. miR-34a is commonly deactivated in human PC cells and transactivated by p53 tumor suppressor protein (Chang et al., 2007; Raver-Shapira et al., 2007). Downregulation of miR-15a (Zhang et al., and 2010), miR-124 (Wang P. et al., 2014) is also associated with proliferation, progression, and metastasis of PC.

Deregulation of miRNAs can be connected with PC, therefore, most of the articles regarding miRNA in PC focused on the potent diagnostic or prognostic use instead of therapeutic implication. Julia S. Johansen et al. screened 38 microRNAs and identified two diagnostic panels holding approximately 80% accuracy, sensitivity, and specificity (Schultz et al., 2014). The expression of miR-191, -21, and -451a derived from serum exosomal was significantly up-regulated in patients with PC, which was served as early diagnostic and progression markers of PC with an accuracy of 60–80% (Goto et al., 2018). In particular, compared with carcinoembryonic antigen (CEA) and carbohydrate antigen 19-9 (CA19-9) termed asstandard and widely-known biomarkers and the detection of PC, circulating miR-25 could also be a promising biomarker for the diagnosis of early-stage PC owing to its high specificity (Deng et al., 2016). Moreover, a clinical trial related to the detection of microRNA-25 for the diagnosis of PC is already in progress in China (Takakura et al., 2019).

As for therapeutic implications, “miRNA mimics” are double-stranded miRNA with suitable chemical modifications, which are often used for miRNA-based anticancer therapies (Rawat et al., 2019). Pierre Cordelier et al. reported the strong reduction of *let-7* microRNA in PC. Restoring *let-7* levels strongly suppressed *KRAS* expression and cell proliferation in Capan-1 cells. However, this could not inhibit the progression of tumor growth (Torrisani et al., 2009). Hsa-microRNA-520h was also proven to target ATP-binding cassette superfamily G member 2 (ABCG2) highly expressed in PC, thus leading to inhibition of cell growth especially migration and invasion in PANC-1 cells (Wang et al., 2010). Furthermore, miR-96 showed the downregulation ability of *KRAS*, resulting in inhibition of cancer cell and tumor growth both *in vitro* and *in vivo* (Yu S. N. et al., 2010). Notably, adenovirus-mediated delivery of miR-143 also exhibited significant inhibition of PANC-1 cancer cells and xenograft tumor growth (Hu et al., 2012). Likewise, restoring miR-150 to the normal level could also dramatically impede the tumor growth of PC cells in a manner of downregulation of MUC4 protein levels (Srivastava et al., 2011).

#### 2.3.2 Targeting Downregulated MicroRNAs

For miRNAs related to PC whose expression is upregulated, oligonucleotides especially antisense oligonucleotides and small molecule inhibitors were reported. A portfolio of antimirR-21 ASO and gemcitabine adopting scFv-functionalized nanoparticle exerted synergistic antitumor effects on PC cells (Li Y. Q. et al., 2017). Another antimiRNA-199a oligonucleotides adopted novel cell-penetrating peptide-based nanocomplexes as the carrier, which significantly inhibited the differentiation of hPSCs and prevented tumor heterospheroids (Schnittert et al., 2017). Anti-microRNA oligonucleotides encapsulated by human serum albumin-assistant nanosystem (EPOC and cholesterol-related liposomes) showed efficient abolishment of microRNA expression (miR-21, miR-221, miR-222), with a strong and synergistic antitumor effect plus chemotherapeutic drug sunitinib (Passadouro et al., 2014). One small molecule azobenzene has also shown specific and efficient inhibition of miR-21 expression *in vitro* (Gumireddy et al., 2008).

### 2.4 Messenger RNA

The carcinogenesis process is always accompanied by the dysregulation of various proteins and this transition requires a high level of mRNA translation. Unlike small molecules and oligonucleotides, mRNA usually consists of hundreds or thousands of nucleotides with a single strand including untranslated regions. Omacetaxine mepesuccinate (homoharringtonine) was approved by the FDA for the treatment of chronic myelogenous leukemia (CML) in 2012. Later, several drugs such as tomivosertib, selinexor, and ribavirin have also entered clinical trials *via* the mechanism of inhibiting mRNA translation (Pal et al., 2019).

Numerous genetic and epigenetic alterations could be detected within PC and correlate with the dysregulation of mRNA translation. For instance, the transcription factor nuclear factor erythroid-derived 2-like 2 (NRF2) is vital to stimulate the PC initiation and maintain proliferation by regulating mRNA translation, which makes the NRF2 ablation a promising strategy for the treatment of PC (Chio et al., 2016). Insulin-like growth factor 2 mRNA binding protein 2 (IGF2BP2), which is capable of binding to various transcripts and regulating RNA functions, is situated at a high level within PDAC, therefore resulting in a poor prognosis. IGF2BP2 could also promote PDAC progression through the straightforward binding of glucose transporter 1 (GLUT1) mRNA (Huang et al., 2019). The downregulation of BAG3, one of the HSP families, is correlated with poor prognostics in PDAC, which could attribute to the suppression of AGO2-related degradation of IL6 mRNA (Li et al., 2019). The pathogenic mechanism research facilitates the druggable and therapeutic strategy but remains at an extreme earlier stage. Notably, Yoshio Sakai designed one PCR-based detecting strategy to monitor the dysregulated genes related to PDAC with a high sensitivity of more than 70% independent of the stage of PDAC (Sakai et al., 2019).

**FIGURE 2:**
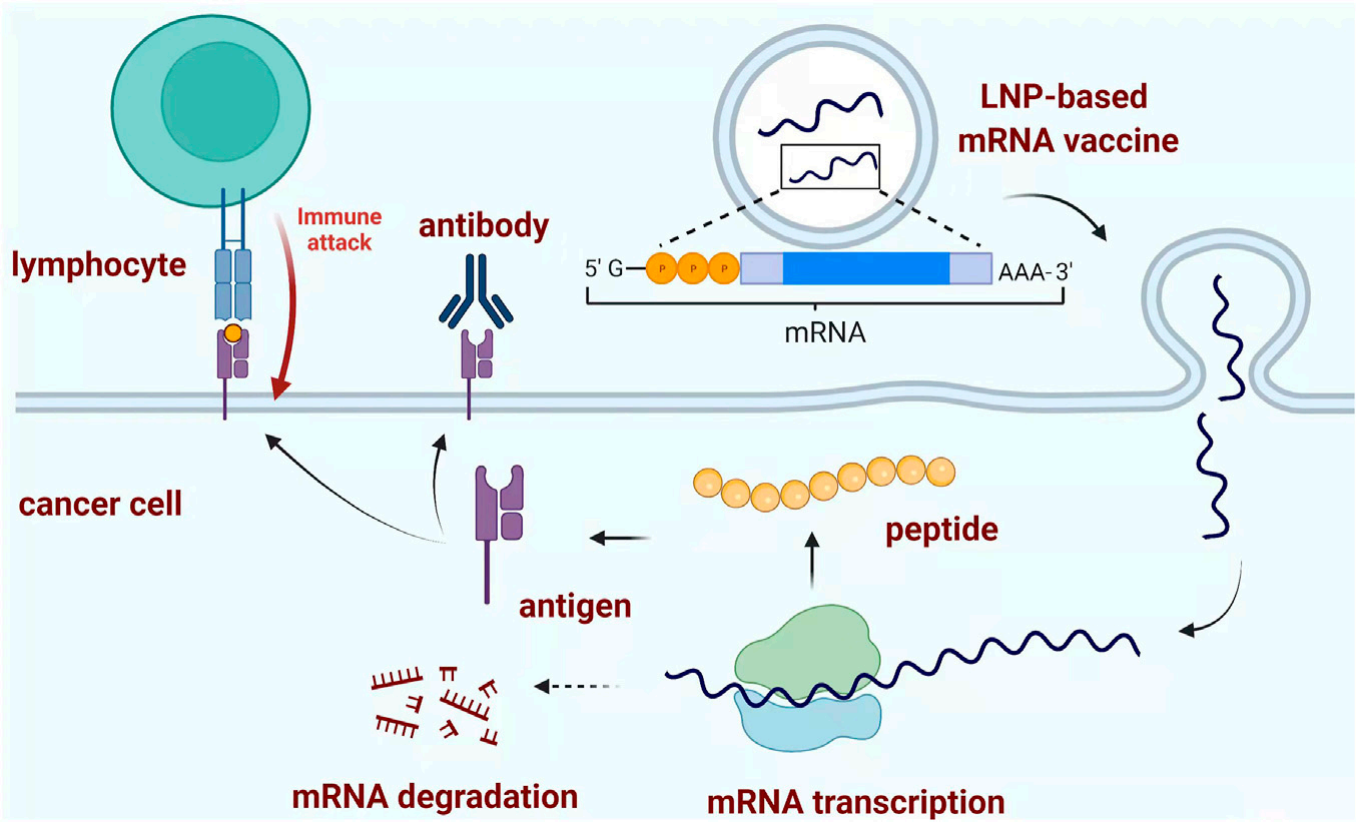
Typical mechanism of mRNA vaccine. LNP-based mRNA vaccine could induce the formation of peptide-based antigen through mRNA transcription as the blueprint. The produced antigen could stimulate the adaptive immune response to destroy the corresponding pathogen or cancer cells. Created with BioRender (License No: HK23PCSH9W).

mRNA vaccines have been effective against multiple cancers but with limited reports against PDAC, the major mechanism for mRNA vaccine is based on the identification of specific tumor antigen, followed by the activation of the immune system to recognize and eliminate cancer cells (Figure 2) (Dorrie et al., 2020). Recently one group has investigated and identified six potential antigens within PDAC patients, which could lay the foundation for the development of anti-PDAC mRNA vaccines (Huang et al., 2021). Another group utilized the combination of antibodies which could specifically bind and neutralize C-C motif chemokine ligand 2 (CCL2) and CCL5 (BisCCL2/5i) and mRNA encoding BisCCL2/5i within a clinical available delivery system. Their results indicated that the BisCCL2/5i mRNA nanoplatforms could dramatically activate sufficient immune response and inhibit cancer growth. Together with PD-1 ligand inhibitor (PD-Li), this combination strategy could achieve significant tumor inhibition and a longer survival time within mouse models (Wang Y. et al., 2021).

## 3 Developing Aptamer-Drug Conjugates of Pancreatic Cancer

Aptamers, usually screened by systematic evolution of ligands by exponential enrichment (SELEX) protocol, contain 20–100 nucleotides with high affinity to the target (Zhou and Rossi, 2017; Rothlisberger and Hollenstein, 2018). The binding affinity largely depends on the three-dimensional structure, including loop, bugle, pseudoknot, G-quadruplex, and kissing hairpin (Keefe et al., 2010; Dunn et al., 2017). Compared with antibodies, aptamers have a much smaller size of 6–30 kDa, preferential penetration, reduced toxicity, lower immunogenicity, and longer shelf life (Table 2) (Zhou and Rossi, 2017; Odeh et al., 2020). At present, eleven aptamers have entered clinical trials, one of which named NOX-A12 was designed for the treatment of metastatic PC (Kaur et al., 2018; Halama et al., 2020). This aptamer targets chemokine ligand 12 (CXCL12), which has been proven to play an important role in PC growth, metastasis, and particular recurrences (De Nigris et al., 2012).

**TABLE 2:** Comparison between antibody and aptamer.

Characteristic	Antibody	Aptamer	Note
Affinity	High	High	Usually own a *K*_D_ value of nanomolar level
Cost	High	Medium	Less costly to manufacture GMP-grade aptamers in large quantities
Manufacturing process	Cell culture (4–6 months)	Chemical synthesis (2–3 months)	—
Immunogenicity	High	Low	Small DNA and RNA aptamers are inherently non-immunogenic
Modification	Usually conjugated with signaling or binding molecule	Versatile	The aptamer can be easily modified during the synthetic process
Size	150–170 kDa (IgG)	12–30 kDa (∼30–80 nucleotides)	Small size aptamer can infiltrate tissues and sometimes cells
Stability	Susceptible to high temperatures and pH changes	Fairly stable at ambient temperature	The aptamer can be refolded if denatured
Specificity	High	High	—

**FIGURE 3:**
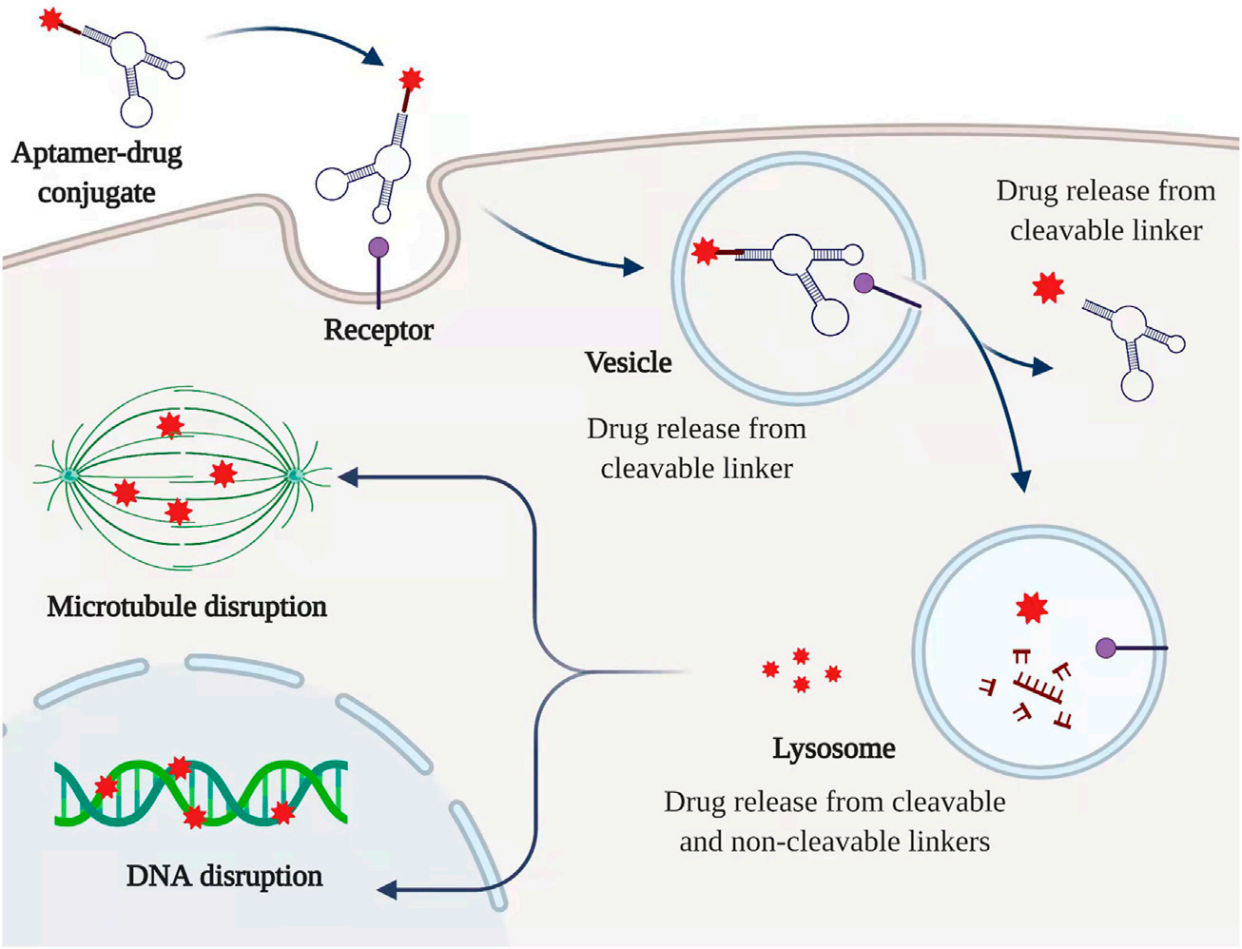
Typical mechanism of ApDC. ApDC could be internalized by a specific receptor which is highly expressed among PC cells. The cytotoxic drugs can be released from cleavable and non-cleavable linkers along with the degradation of the aptamer. Created with BioRender (License No: EQ23P90T7N).

Various aptamers have been investigated or developed upon the antitumor activity. For example, Luigi E. Xodo et al. found a new quadruplex oligonucleotide with TINA modification based on the promoter of the human *KRAS* gene. This new G-quadruplex motif lifted the *T*_m_ value and indicated a significant antiproliferative activity within PANC-1 cancer cells (Cogoi et al., 2009). Another 2′-fluoropyrimidine modified RNA aptamer (P12FR2) designed against human overexpressed pancreatic adenocarcinoma up-regulated factor (PAUF) with a high binding affinity of 77 nM was also reported, which displayed strong inhibition activity in PANC-1 cells as well as up to 60% inhibition of tumor shrinkage *via* intraperitoneal injection of P12FR2 (Kim et al., 2011). However, in most cases, aptamers are adopted as the specific cargo to form ApDCs to deliver different kinds of drugs to their target. In terms of exerting mechanism, the conjugated drug is supposed to release with the break of a specific linker upon the accumulation into aptamer’s target which is usually highly expressed on the surface of cancer cells (Figure 3).

### 3.1 RNA Aptamers-Based Conjugates

The upregulation of some specific protein receptors is common in PC cells, which enables the specific binding and internalization of aptamer together with the desired cargo as the therapeutic drug (Table 3). Rebekah R. White group developed a nuclease resistant RNA aptamer targeting EGFR for the delivery of gemcitabine-containing polymers into epidermal growth factor receptor (EGFR)-expressing cells, which, as a result, showed strong inhibition of cell proliferation (Ray et al., 2012). Alkaline phosphatase placental-like 2 (ALPPL2) is also a putative biomarker for PC. Dong-ki Lee et al. identified a nuclease-resistant RNA aptamer SQ2 with a specific affinity to ALPPL2 and made use of this aptamer to deliver 5-fluorouracil to exert inhibition of cell proliferation in Capan-1 PC cells (Dua et al., 2015). Another oligonucleotide chimera, composed of an antitransferrin receptor RNA aptamer as the targeting carrier and a DNA decoy oligonucleotide as an NF-κB inhibitor showed increased therapeutic efficacy of Doxorubicin with selectivity in pancreatic tumor cells (Porciani et al., 2015). P19, as another PC-specific RNA aptamer, was also used to conjugate active metabolites of neoadjuvant gemcitabine (dFdCMP) and 5FdU (5FdUMP) into the strand, resulting in over 50% inhibition of cell proliferation in PANC-1 and AsPC-1. The Monomethyl auristatin E (MMAE) drug was further attached to modified P19 aptamer, which significantly increased specific uptake of the aptamer-drug conjugate in tumor cells (Yoon et al., 2016a; Yoon et al., 2017a). Similarly, Monomethyl auristatin F (MMAF) was conjugated to an anti-EGFR aptamer to form E07, which could serve as one alternative in terms of specific delivery into PC cancer cells including PANC-1, MIA PaCa-2, and BxPC-3 (Kratschmer and Levy, 2018). Chung-Hsuan Chen et al. developed Ap52 aptamer targeting tumor-specific MAGE-A3_111-125_ peptide antigen and further phosphorothioate-modified Ap52 (ThioAp52)-mediated delivery of anticancer drug indicated the ThioAp52-Dox complex has a specific selectivity against PC cells when compared with naked Dox (Wang C. Y. et al., 2021). John J Rossi et al. used blind cell SELEX and successfully developed one P15 aptamer specifically bound to the cell surface vimentin of human pancreatic adenocarcinoma cells. Meanwhile, this P15 aptamer was associated with reduced matrix metallopeptidase 3 (MMP3) expression highly related to tumor cell invasion (Yoon et al., 2017b).

**TABLE 3:** Aptamer-mediated targeting strategies under current investigation for the treatment of PC.

Aptamer (Length of Nucleotides)	Target and binding affinity (*K*_D_)	Payload	Linker	IC_50_	Tumor shrinkage *in vivo*	References
SQ2 (28)	Alkaline phosphatase placental-like 2 (ALPPL2), 20 nM	5-FU	3′-conjugation	Capan-1 (micromolar)	—	Dua et al. (2015)
C2C (43)	Transferrin, 102 nM	DOX and NF-κB decoy	NUPACK (DOX) Disulfide bond (NF-κB decoy)	NA^a^	—	Porciani et al. (2015)
P1/P19 (87)	PANC-1 (P19, 13.07 nM) (P1, 12.69 nM)	C/EBPα-saRNAs	*STICK* ^b^	NA	∼30%	Yoon et al. (2016a)
Tp19 (27)	PANC-1, 8.7 nM	MMAE/DM1	*STICK*	NA	—	Yoon, et al., 2016b; Yoon, et al., 2017a
AP1153 (49)	Cholecystokinin B receptor, 0.015 nM	CPNSPs	PEG	—	—	Clawson et al. (2017)
E07 (47)	Epidermal growth factor receptor (EGFR), 51 nM	Monomethyl auristatin E (MMAE)	Cathepsin-cleavable (MC-VC-PAB)	MIA PaCa-2 (130 nM) PANC-1 (55 nM)	—	Kratschmer and Levy, (2018)
AP52 (52)	MAGE-A3_111-125_, 13.07 nM	DOX	Incorporation	NA	—	Wang C. Y. et al. (2021)
AS1411 (26)	Nucleolin, 16.36 nM	Triptolide	PEG-PDLLA	MIA PaCa-2 (∼75 nM)	—	Wang et al. (2016)
		Gemcitabine	Incorporation	AsPC-1 (204 nM) Capan-1 (69 nM) MIA PaCa-2 (284 nM)	42.5%	Park et al. (2018)
tTR14 (22)	Transferrin, 0.022 nM	C/EBPa-saRNA	*STICK*	NA	Up to 85%^c^	Yoon et al. (2019)

a Inhibition data were reported but no exact IC_50_ value presented in the primary literature.

b A “sticky” sequence was placed between aptamer and C/EBPa-saRNA, followed by annealing both strands.

c This value was achieved by Ttr14-C/EBPa-saRNA conjugate supplemented with gemcitabine.

Other P19- and P1-CCAAT/enhancer-binding Protein α (C/EBPα)-saRNA conjugates also indicated strong suppression of PANC-1 cell proliferation and reduced tumor size up to 30% with no observed toxicity in gemcitabine-resistant AsPC-1 cells mouse-xenografts (Yoon et al., 2016b). The C/EBPα-saRNA could significantly inhibit tumor growth *in vivo*. Furthermore, this C/EBPα-saRNA was conjugated to another TR 14 RNA aptamer targeting transferrin with a highly-potent affinity of 0.01317 nM. Even though the TR 14 RNA aptamer was truncated to only 22 nucleotides (tTR 14) it could show comparable characteristics. The potent anti-tumor activity regarding tumor shrinkage up to the 85% in advanced PDAC mouse model could be observed after administration of tTR 14-C/EBPα-saRNA conjugate supplemented with gemcitabine, while gemcitabine alone could reduce the tumor volume ∼70% (Yoon et al., 2019).

### 3.2 DNA Aptamers-Based Conjugates

AS1411 is an aptamer targeting nucleolin which is usually overexpressed by PC cancer cells. Won Jun Kang et al. incorporated gemcitabine into AS1411 to form APTA-12, resulting in improved *in vitro* cytotoxicity and inhibition of tumor growth in Capan-1 tumor-bearing mice (Park et al., 2018). Weihong Tan et al. identified an ssDNA aptamer termed XQ-2d based on cell-SELEX technology and demonstrated the XQ-2d mediated delivery of doxorubicin showed increased uptake by a variety of cancers including PC (Wu et al., 2019).

In addition, nanoparticles are often taken as efficient codelivery vehicles together with aptamers in PC. Dual drugs containing gemcitabine and curcumin conjugated to AS1411 and magnetic PLGA nanoparticles exhibited strong inhibition of cell growth in PANC-1 and MIA PaCa-2 (Sivakumar et al., 2013). Triptolide, as a superior anticancer agent was also conjugated to AS1411 followed by the preparation of AS1411-PEG-PDLLA micelle loading TP (AS-PPT). AS-PPT exhibited more antitumor activity than triptolide and showed increased overall survival in mouse models compared with the clinical anticancer drug Gemzar (Wang et al., 2016). One DNA aptamer termed AP1153 was successfully selected and exhibited a highly potent *K*_d_ value of 15 pM to G-protein-coupled cholecystokinin B receptor (CCKBR). AP1153 was further conjugated to the surface of nanoparticles and this delivery system could significantly penetrate pancreatic tumors in animal models (Clawson et al., 2017).

Notably, Chen Jiang et al. conjugated ECM-derived tenascin-C targeting aptamer (GBI-10) to a stroma-permeable cell-penetrating peptide (CPP). In the stroma, tenascin-C can detach and the exposed CPP can facilitate pancreatic tumor penetration endocytosis. The intracellular high redox environment provided the potential to trigger the controlled release of chemotherapy drugs. The *in vitro* cytotoxicity was relatively mild; however, the *in vivo* antitumor efficacy was excellent together with decreased systemic toxicity (He et al., 2018).

## 4 Discussion and Future Perspectives

Up to date, patients with advanced and metastatic PDAC only own a median survival from 6 to 12 months (Conroy et al., 2018). Cytotoxic chemotherapy gemcitabine, together with nab-paclitaxel and FOLFIRINOX make up the standard and prime treatment of PC. However, chemotherapy usually leaves patients suffering from various adverse effects and poor prognoses. Many studies have paid much attention to the development of small molecules inhibitors for PDAC treatments. For example, one tyrosine kinase receptor inhibitor pazopanib showed promising results in a phase II study for patients with advanced, well-differentiated neuroendocrine tumors (NETs) (Phan et al., 2015). However, NETs are scarce (<10%) and require further phase III study. Some other studies have focused on the inhibition of vital signaling pathways related to PDAC, such as focal adhesion kinase (FAK) inhibitor, signal transducer, and activator of transcription 3 (STAT3). Although FAK inhibitor against PDAC has entered clinical trial, drug resistance and a lack of deep understanding of the mechanism could still hinder further development (Jiang et al., 2020). Notably, one group developed one dendrimer nanocarrier to deliver doxorubicin, resulting in better responses against 35 major PC cell lines than free drug, which might offer an insight into the nanosystem-aided therapy (Liu et al., 2021).

The overall progress is usually heavily-limited compared with the patient survival relative to current chemotherapy (Sun et al., 2020). This lack of enhancement could attribute to the superabundant extracellular matrix with desmoplastic fibrotic stroma accounting for 80% of tumor volume, which in turn limits the effective delivery of therapeutical chemotherapy agents into pancreatic tumors and correlates with the poor prognosis (Kota et al., 2017). Thus, in recent years, many researchers have concentrated on the characteristics of the tumor microenvironment especially in the stroma environment and inside cellular communication led by CAFs, PSCs, muscle fibroblasts, and immune cells. Unfortunately, PEGPH20, sonic hedgehog inhibitors and matrix metalloproteinase (MMP) inhibitors targeting and reshaping the tumor stroma microenvironment followed by the drug administration have shown limited clinical efficacy (Vennin et al., 2018; Ho et al., 2020). Recently, targeting Pin1 has been demonstrated to completely eliminate or sustain remissions of aggressive PDAC in diverse model systems by firstly remodeling the original tumor microenvironment into a less desmoplastic and immune responsive tumor microenvironment followed by antiPD-1 and gemcitabine therapy (Koikawa et al., 2021). On the other hand, current preclinical models of PC are poor and could not reflect reality, which usually leads to failure in clinical studies. Reducing stromal collagen and hyaluronan production and targeting the vitamin D receptors overexpressed in the stroma to facilitate the chemotherapy administration were also blocked by the same limitation. In this way, the desmoplastic fibrotic stroma within the tumor microenvironment is one unavoidable obstacle that remains to be settled and should be considered as a priority when confronted with the drug discovery of PC.

Compared with conventional drugs, nucleic acid drugs afford a versatile approach to treat a wider range of diseases from the underlying causes. The delivery system is vital in enhancing the druggability of nucleic acid drugs and there are already some clinical examples based on *N*-acetylgalactosamine (GalNAc) conjugation, lipid nanoparticles (LNPs), and adeno-associated virus (AAV) vector systems (Kulkarni et al., 2021; Van Den Berg et al., 2021; Vargason et al., 2021). Unfortunately, there is no clinically available therapeutic RNAi for PC up to date. The point mutations of the *KRAS* gene could be detected in over 90% of human PCs and the introduction of anti-*KRAS* ASO and siRNA have shown ideal knock-out efficiency and tumor inhibition in orthotopic mouse models, but few clinical trials could be identified to give fundamental support for the treatment of PC. ASOs targeting cell proliferation factors including XIAP, protein Kinase A (PKA), cell signaling (HRAS), resistance to chemotherapy (Hsp27), and cancer stroma (TGFβ2) all failed to show antitumor effects in clinical trials (Yamakawa et al., 2019). Some other siRNAs targeting mutated *KRAS* in combination with gemcitabine treatment showed potential efficacy in PC patients in early-phase trials but no more progress has been reported (Yamakawa et al., 2019). For the FDA-approved siRNA/ASO drugs, all candidates are used for rare disease treatment and most of them are highly modified because of the highly-charged characteristic and the serum instability. The utility of highly optimized chemical modifications, conjugation to multiple ligands, and nanoparticle carriers significantly have improved the delivery efficiency. Nevertheless, due to the sophisticated tumor microenvironment and the desmoplastic fibrotic stroma of PC, it becomes much more complex to efficiently deliver RNAi therapeutics into the pancreatic tumors. Therefore, the selection and development of best-fit chemical modifications, and delivery systems capable of overcoming the inherent shortcomings and potential targets for RNAi within PC and even stroma largely contribute to the relevant drug discovery (Vetvicka et al., 2021). mRNA vaccines and translation inhibitors as well as therapeutical vaccines represent novel and promising strategies against PC, but more preclinical and clinical evidence necessary for the drug discovery of PC are still required.

Compared with antibodies, aptamers have a smaller molecular weight but own considerable affinity owing to the complex tertiary folded structures, which can be chemically synthesized and modified with more availability and flexibility enabling the long shelf life and low immunogenicity characteristics favorable for clinical implications and precision medicine. Since the first approval of aptamer drug pegaptanib for the treatment of wet-age-related macular degeneration, an increasing number of aptamer therapeutics for oncology are in clinical trials (Morita et al., 2018). ApDCs, as one combination of aptamers and cytotoxic molecules, have shown promising results in preclinical models against PC. It has been almost one decade since the first model of the aptamer-drug conjugates or chimeras while ADCs have already entered third generations over two decades since the first approval of Mylotarg (Beck et al., 2017; Xuan et al., 2018). The overall drug research and development of ApDCs have largely lagged behind ADCs and no ApDCs have gone far to clinical trials. The screening and discovery of new high-affinity and specificity targeting aptamers towards PC, together with the combination of reshaping or remodeling the pancreatic tumor microenvironment, might hold promise for the future treatment of PC.

## Glossary

**AAV**: Adeno-associated virus
**ApDC**: Aptamer-drug conjugate
**5-FU**: 5-Fluorouracil
**ABCG2**: ATP-binding cassette super-family G member 2
**ADC**: Antibody-drug conjugate
**AGO2**: Argonaute RISC Catalytic Component 2
**ALPPL2**: Alkaline phosphatase placental-like 2
**AP1153**: One DNA aptamer targeting CCKBR
**APTA-12**: Gemcitabine-incorporated AS1411
**AS1411**: One DNA aptamer targeting nucleolin
**ASO**: Antisense oligonucleotide
**AsPC-1**: Human pancreatic tumor cell line
**AS-PPT**: AS1411-PEG-PDLLA micelle
**BAG3**: BAG family molecular chaperone regulator
**Bcl-2**: B-cell lymphoma 2
**Bcl-xL**: B-cell lymphoma-extra large
**BCPV**: Biodegradable charged polyester-based vector
**BxPC-3**: Human pancreatic adenocarcinoma cell line
**C/EBP** *α*: CCAAT/Enhancer-binding Protein *α*
**CA19-9**: Carbohydrate antigen 19-9
**CAF**: Cancer-associated fibroblast
**Capan-1**: Human pancreatic ductal adenocarcinoma cell line
**CCKBR**: G-protein-coupled cholecystokinin B receptor
**CCL2**: C-C motif chemokine ligand 2
**CCL5**: C-C motif chemokine ligand 5
**CCR4-NOT**: Carbon catabolite repression 4–negative on TATA-less
**CD8^+^ T cells**: Cytotoxic T lymphocytes cells
**CEA**: Carcinoembryonic antigen
**CML**: Chronic myelogenous leukemia
**COVID-19**: Coronavirus Disease 2019
**COX-2**: Cyclooxygenase-2
**CPNSPs**: Coprinopsis psychromorbida
**CPP**: Cell-penetrating peptide
**cRGD**: Cyclic arginylglycylaspartic acid
**CSF-1**: Colony stimulating factor 1
**CSF-1R**: Colony stimulating factor 1 receptor
**CXCL10**: C-X-C motif chemokine ligand 10
**CXCL12**: Chemokine ligand 12
**dFdCMP**: Gemcitabine monophosphate
**DM1**: Mertansine
**DOX**: 4-Substituted-2,5-dimethoxyamphetamines
**ECM**: Extracellular matrix
**EGFR**: Epidermal growth factor receptor
**FAK**: Focal adhesion kinase
**FDA**: U.S. food and drug administration
**FOLFIRINOX**: Folinic acid, fluorouracil, irinotecan and oxaliplatin
**GalNAc**: *N*-acetylgalactosamine
**GBI-10**: One DNA aptamer targeting tenascin-C
**GLUT1**: Glucose transporter 1
**HaP-T1**: N-nitrosobis-(2-hydroxypropyl) amine-induced hamster pancreatic tumor
**Hsp**: Heat shock protein
**IC_50_**: Half-maximal inhibitory concentration
**IGF2BP2**: Insulin-like growth factor 2 mRNA binding protein 2
**IL-6**: Interleukin 6
***KRAS***: Kirsten rat sarcoma virus
***let-7***: Lethal-7 gene
**LNP**: lipid nanoparticle
**MAGE-A3**: Melanoma-associated antigen 3
**MIA PaCa-2**: Hypotriploid human pancreatic cancer cell line
**miRNA**: microRNA
**MMAE**: Monomethyl auristatin E
**MMAF**: Monomethyl auristatin F
**MMP**: Matrix metalloproteinase
**MMP3**: Matrix metallopeptidase 3
**mRNA**: Messenger RNA
**MUC4**: Mucin 4
**NETs**: Neuroendocrine tumors
**NF-κB**: Nuclear factor-κB
**NGF**: Nerve growth factor
**NRF2**: Nuclear factor erythroid-derived 2-like 2
**P12FR2**: 2′-fluoropyrimidine modified RNA aptamer
**PAN2-PAN3**: Poly (A)-nuclease 2-poly (A)-nuclease 3
**PANC-1**: Human pancreatic ductal cancer cell line
**PAUF**: Pancreatic adenocarcinoma up-regulated factor
**PC**: Pancreatic cancer
**PCR**: Polymerase chain reaction
**PD-1**: Programmed cell death protein 1
**PDAC**: Pancreatic ductal adenocarcinoma
**PDCD4**: Programmed cell death protein 4
**PD-Li**: Programmed cell death ligand inhibitor
**PDLLA**: Poly (DL-lactide)
**PLK1**: Polo-like kinase 1
**PEG**: Polyethylene glycol
**PEGPH20**: Pegvorhyaluronidase alfa
**PI3K-γ**: Phosphatidylinositide 3-kinase γ
**PK-45H**: Human pancreatic carcinoma cell line
**PKA**: Protein Kinase A
**PLGA**: Poly (lactic-co-glycolic acid)
**PSC**: Pancreatic stellate cell
**pSUPER**: Suppression of endogenous RNA
**PTEN**: Phosphatase and tensin homolog
**RCT**: Randomized controlled trial
**RIG-I**: Retinoic acid-inducible gene I
**RISC**: RNA-induced silencing complex
**RNAi**: RNA interference
**RNase H1**: Ribonuclease H1
**saRNA**: Small activating RNA
**SELEX**: Systematic evolution of ligands by exponential enrichment
**siRNA**: Small interfering RNA
**STAT3**: Signal transducer and activator of transcription 3
**SUIT-2**: Human pancreatic cancer cell line from liver metastasis
**T3M4**: Human pancreatic carcinoma cell line
**TGF**: Transforming growth factor
**TIME**: Tumor immune microenvironment
**TP53**: Tumor protein 53
**TrkA**: Tropomyosin receptor kinase A
**tTR 14**: An RNA aptamer targeting transferrin
**VEGF**: Vascular endothelial growth factor
**XIAP**: X-linked inhibitor of apoptosis protein
**XQ-2d**: One DNA aptamer targeting PDAC
